# Pregnancy confirmed after controlled ovarian stimulation for infertility: A case report

**DOI:** 10.1097/MD.0000000000027140

**Published:** 2021-09-17

**Authors:** Meiyan Jiang, Chong Wang, Xiaoyang Fei, Zhenyun Lin

**Affiliations:** Center of Reproductive Medicine, Hangzhou Women's Hospital, Hangzhou, China.

**Keywords:** controlled ovarian stimulation, embryo transfer, in vitro fertilization, pregnancy

## Abstract

**Rational:**

Induction of ovarian stimulation by use of the gonadotropin-releasing hormone agonist (GnRHa) long protocol in the luteal phase is a common practice and results in stable pregnancy and live births; it is often used in patients with normal ovarian function. Some patients with normal ovulation may be pregnant before ovulation induction, which can be easily confirmed by asking the patient about cessation of menstruation. However, some pregnancy complications may cause vaginal bleeding along with normal menstrual blood loss; in such a situation, hormone levels can often mirror that seen in pituitary down-regulation and the value of β-HCG may be less than 5 mIU/mL. Under these conditions, the physician might start the cycle of ovarian stimulation. During ovarian stimulation, the increase in β-HCG can cause premature luteinization and follicle maturation disorder, and poor embryo quality, which can easily be overlooked. In this study, we report a case of pregnancy at the end of controlled ovarian stimulation induced by GnRHa long protocol in the luteal phase, followed by follicle maturation disorder and poor embryo quality. This case provided a reference and served as a cautionary note that could perhaps obviate occurrence of similar cases.

**Patient concerns:**

A 30-year-old woman with a diagnosis of unexplained infertility was scheduled for in vitro fertilization embryo culture (IVF) at our clinic. Pregnancy was confirmed at the end of controlled ovarian stimulation, which was followed by follicular maturation disorder and poor embryo quality.

**Diagnosis:**

The patient with a diagnosis of unexplained infertility was scheduled for IVF at our clinic.

**Interventions:**

Oocyte retrieval was still arranged for her after confirmation of pregnancy. As per the β-HCG level and the trans-vaginal ultrasound examination findings, we considered 2 possibilities: an adverse intrauterine pregnancy or extra-uterine pregnancy. Therefore, we decided to terminate the pregnancy; hence, 50 mg/d of mifepristone was given for 2 days, combined with 200 μg misoprostol.

**Outcomes:**

Elevated β-HCG level had an adverse effect on maturation and fertilization of oocytes, and even embryo quality.

**Conclusion:**

Once pregnancy is confirmed, ovulation induction should be terminated as soon as possible.

## 1 Introduction

Gonadotropin-releasing hormone agonist (GnRHa) long protocol in the luteal phase is a classical protocol commonly used in current practice, that results in stable pregnancy and live birth rates; it is often used in patients with normal ovarian function. Some patients with normal ovulation may be pregnant before the induction of ovulation; this can be easily confirmed by cessation of menstruation. However, some complications in pregnancy can cause vaginal bleeding along with loss of normal amount of menstrual blood; in such a situation, hormone levels can often mirror that seen in pituitary down-regulation. Under this condition, the physician might start the cycle of ovarian stimulation. During stimulation, the rise in β-HCG can cause premature luteinization of follicles, hinder maturation of follicles, and lead to poor embryo quality, which can easily be overlooked by the treating physician. Herein, we report a case of pregnancy at the end of controlled ovarian stimulation induced by GnRHa long protocol in the luteal phase, which was followed by follicle maturation disorder and poor embryo quality, thereby providing a reference that can serve as a cautionary signal helping perhaps obviate occurrence of similar cases.

## 2 Case presentation

A 30-year-old woman (weight: 59 kg; body mass index: 24.6) with a diagnosis of unexplained infertility was scheduled for in vitro fertilization embryo culture (IVF) at our clinic. She failed to conceive after 2 cycles of artificial insemination at our clinic. Her menstrual cycle was 30 to 35 days, lasting 3 to 4 days. Her basal serum follicle-stimulating hormone (FSH), luteinizing hormone (LH), and estradiol (E2) levels were 7.21 IU/L, 3.17 IU/L, and <20 pg/mL, respectively. The numbers of antral follicles were 8 per ovary. In the mid-luteal phase, the patient was administered 0.1 mg/d of short-acting GnRHa (Decapeptyl, Ferring, Kiel, Germany) for pituitary down-regulation in order to initiate IVF. After 14 days, serum FSH was 3.05 IU/L, LH was 0.63 IU/L, progesterone (P) was 1.23 ng/mL, and E2 was 35.0 pg/mL; the value of β-HCG was less than 5 mIU/mL (Table 1). Trans-vaginal ultrasound examination showed that there were 16 or 17 follicles with an average diameter <5 mm, and the thickness of the endometrium was <5 mm. During pituitary down-regulation, vaginal bleeding lasted for 4 days. After confirmation of pituitary down-regulation, administration of recombinant FSH (Gonal-F; Merck–Serono, Eysins, Switzerland) for ovarian stimulation was started at an initial dose of 150 IU/d. On day 6, the average diameter of the follicles was <9 mm, and serum LH was 0.98 IU/L, P was 1.23 ng/mL, and E2 was 324 pg/mL; hence, 37.5 IU of human menopausal urinary gonadotropin (HMG) (Livzon Pharmaceutical, Shanghai, China) was added for 2 days. On day 8, ultrasound examination showed that there were 10 follicles with average diameter >10 mm, and the largest was follicle was 11 mm. Serum LH was 1.44 IU/L, P was 1.24 ng/mL, and E2 was 944 pg/mL; therefore, 37.5 IU/d of HMG and 150 IU/d of FSH were administered for stimulation of follicular growth. On day 10, serum LH was 0.73 IU/L, P was 2.04 ng/mL, and E2 was 2542 pg/mL; level of P was higher than before. The speed of follicular growth was normal; thus, 37.5 IU of HMG and 150 IU of FSH were administered from day 10 through 12. On day 13, the average diameter of 8 follicles was >18 mm and the diameter of 5 of them was 14 to 17 mm. Serum *P* value increased to 9.64 ng/mL, LH was <0.2 IU/L, and E2 was 5446 pg/mL. Because the serum P was higher than before, β-HCG was detected at a level of 275.1 mIU/mL. Induction with 4000 IU of HCG (Profasi, Serono) was done and oocyte retrieval was performed 36 hours after HCG induction. Routine luteal support (20 mg progesterone orally, twice daily) was given after oocyte retrieval.

**Table 1:** Laboratory data.

Date/Cycle	12.19/20	1.2	1.7	1.9	1.11	1.13	1.14	1.17	1.19	1.21	1.29
Date of Gn		1	6	8	10	12	13				
Follicle of left ovary (mm)	6^∗^8	6^∗^8	8^∗^10	10.5^∗^29.5^∗^6	13^∗^112.^∗^39^∗^3	17.5^∗^315^∗^311.5^∗^4	21^∗^318^∗^217^∗^3				
Follicle of right ovary (mm)	6^∗^7	6^∗^8	9^∗^8	11^∗^39.5^∗^3	15.5^∗^112^∗^5	21^∗^115^∗^212^∗^2	26^∗^120^∗^214.5^∗^2				
GnRHa (mg)	0.1	0.05	0.05	0.05	0.05	0.05					
FSH (IU)		150	150	150	150	150					
HMG (IU)			37.5	37.5	37.5	37.5					
HCG (IU)							4000				
E2 (pg/mL)		35	324	944	2542	>4870	5446				
P (ng/mL)		1.23	1.23	1.24	2.04	5.79	9.46				
LH (mIU/mL)		0.63	0.98	1.44	0.73	<0.2	<0.2				
Mifepristone (mg)									50		
Misoprostol (ug)										200	
β-HCG (mIU/mL)		<5					275.1	290.4	288.2		0.7
trans-vaginal ultrasound								the gestational sac was not seen inside or outside of the uterus			

E2 = estradiol, FSH = follicle-stimulating hormone, Gn = gonadotropin, GnRHa = gonadotropin-releasing hormone agonist, HCG = human chorionic gonadotrophin, HMG = human menopausal urinary gonadotropin, LH = luteinizing hormone, P = progesterone.

**Figure 1:**
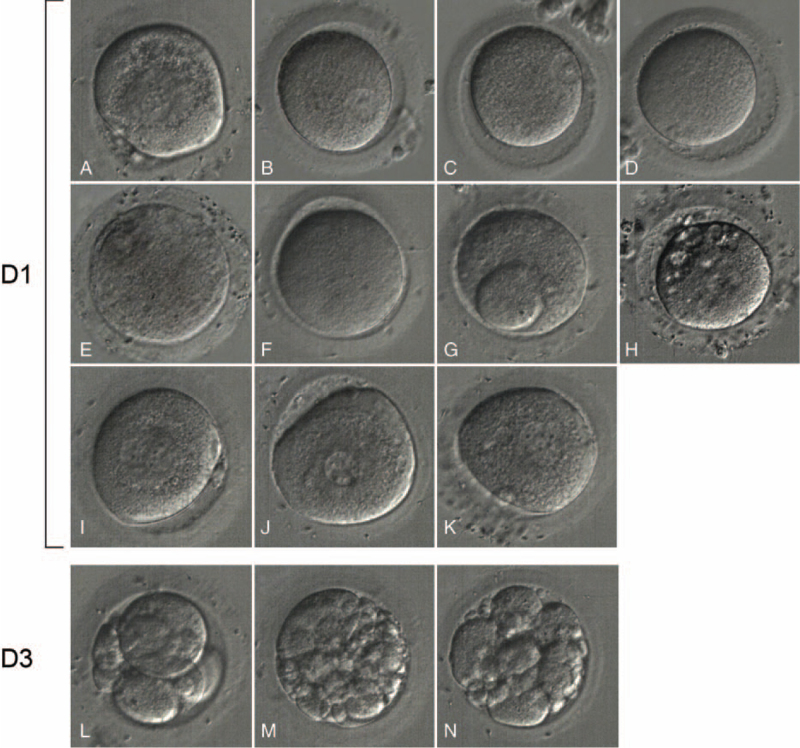
The images of in vitro fertilized oocytes. D1: a, 3PN; b–c, GV; d–f, MI; g, giant polar body oocyte; h–k, 2PN; D3: i, j, k developed to l, m, n; others were degenerated.

On the first day after oocyte retrieval, 4 oocytes had 2 pro-nuclei, one had 3 pro-nuclei, 3 were metaphase I oocytes, 2 were germinal vesicle oocytes, and one had a giant polar body. On day 3 of insemination, 1 embryo evaluated by Gardner's criteria^[1]^ as 7CIII was frozen; another 2 embryos were grade IV at the four-cell stage and continued to be cultured to day 5, but no blastocysts were obtained (Fig. 1).

On the first day after egg retrieval, the serum β-HCG was 290.4 mIU/mL, and on trans-vaginal ultrasound examination, the gestational sac was not seen inside or outside of the uterus. However, after 2 days, β-HCG was only 288.2 mIU/mL. As per the level of β-HCG, there were currently 2 possibilities: an adverse intrauterine pregnancy or extra-uterine pregnancy. Therefore, we decided to terminate the pregnancy; 50 mg/d of mifepristone was given for 2 days, combined with 200 μg misoprostol. β-HCG was checked again and was found to be decreased to 0.7 mIU/mL after 10 days. The ethics committee of Hangzhou Women's Hospital approved this study. In addition, the patient has signed the consent form for publication of this case.

## 3 Discussion and conclusion

LH is essential for providing the androgen substrate for estrogen synthesis, and an LH surge is required for final follicular maturation and ovulation.^[2]^ Hillier et al^[3]^ proposed the existence of a therapeutic window for LH during ovarian stimulation. According to this, there is not only a threshold requirement for LH to guarantee follicular development but also a ceiling level beyond which LH might inhibit the proliferation of granular cells and induce luteinization of follicles before ovulation, resulting in impaired oocyte and embryo quality.^[3–5]^ Some studies have suggested that LH supplementation has a beneficial effect on the maturation and fertilization of oocytes in patients who are undergoing pituitary down-regulation with significant serum LH suppression.^[6–8]^ However, it has been consistently reported that high LH levels during the follicular phase are associated with poor oocyte and embryo quality, with a negative impact on IVF outcome.^[9,10]^ Nonetheless, there is no consensus about the specific threshold of the LH window during follicular development.

In assisted reproduction, because of its structural similarity and similarity in biological action with LH, HCG acts on the same receptor and has been used to mimic the endogenous LH surge.^[11]^ Moreover, HCG has a slower plasma metabolic clearance and may be more effective than LH. In our case, there was continuous secretion of endogenous β-HCG from trophoblastic tissue. Therefore, the high serum HCG level which was equivalent to high level of LH had an adverse effect on the maturation and fertilization of oocytes, and even embryo quality. Therefore, once pregnancy is confirmed, ovulation induction should be terminated as soon as possible.

## Acknowledgments

The authors are grateful to the doctors and staff who were involved in this work.

## Author contributions

**Conceptualization:** Zhenyun Lin.

**Data curation:** Chong Wang.

**Writing – original draft:** Meiyan Jiang.

**Writing – review & editing:** Xiaoyang Fei.
